# Molecular and Functional Characterization of GR2-R1 Event Based Backcross Derived Lines of Golden Rice in the Genetic Background of a Mega Rice Variety Swarna

**DOI:** 10.1371/journal.pone.0169600

**Published:** 2017-01-09

**Authors:** Haritha Bollinedi, Gopala Krishnan S., Kumble Vinod Prabhu, Nagendra Kumar Singh, Sushma Mishra, Jitendra P. Khurana, Ashok Kumar Singh

**Affiliations:** 1 Division of Genetics, ICAR-Indian Agricultural Research Institute, New Delhi, Delhi, India; 2 ICAR-National Research Centre on Plant Biotechnology, Pusa Campus, New Delhi, Delhi, India; 3 Department of Plant Molecular Biology, University of Delhi, South Campus, New Delhi, Delhi, India; National Institute of Plant Genome Research, INDIA

## Abstract

Homozygous Golden Rice lines developed in the background of Swarna through marker assisted backcross breeding (MABB) using transgenic GR2-R1 event as a donor for the provitamin A trait have high levels of provitamin A (up to 20 ppm) but are dwarf with pale green leaves and drastically reduced panicle size, grain number and yield as compared to the recurrent parent, Swarna. In this study, we carried out detailed morphological, biochemical and molecular characterization of these lines in a quest to identify the probable reasons for their abnormal phenotype. Nucleotide blast analysis with the primer sequences used to amplify the transgene revealed that the integration of transgene disrupted the native *OsAux1* gene, which codes for an auxin transmembrane transporter protein. Real time expression analysis of the transgenes (*ZmPsy* and *CrtI*) driven by endosperm-specific promoter revealed the leaky expression of the transgene in the vegetative tissues. We propose that the disruption of *OsAux1* disturbed the fine balance of plant growth regulators *viz*., auxins, gibberellic acid and abscisic acid, leading to the abnormalities in the growth and development of the lines homozygous for the transgene. The study demonstrates the conserved roles of *OsAux1* gene in rice and *Arabidopsis*.

## Introduction

Rice (*Oryza sativa* L.) is one of the three major food crops of the world, vital for the survival of more than half of the world’s population. Globally ~480 million metric tons of milled rice is produced annually, 80% of which is produced on small farms, primarily to meet family needs. Developing countries in Asia are heavily reliant on rice for their dietary caloric supply with about 90 percent of the world’s rice produced and consumed in the six Asian countries (China, India, Indonesia, Bangladesh, Vietnam and Japan). From an area of 44 mha, India produces over 144 million metric tons of paddy-rice and stands as second largest producer globally, next only to China [1].

Inherently, rice endosperm lacks provitamin A, leading to severe vitamin A deficiency among rice eating population in at least 26 countries of Asia, Africa and Latin America [2, 3]. Therefore, enriching the rice endosperm with provitamin A carotenoids through biofortification is a viable and complementary intervention to tackle this menace [4, 5]. Since, rice gene pool lacks the genetic variation for synthesis of pro-vitamin A in endosperm, there is no scope for its improvement through breeding. Genetic engineering overcomes this limitation and opens the avenues for transferring the desirable gene(s) across the sexual barriers.

The complete characterization of the genes encoding various enzymes involved in the carotenoid biosynthetic pathway has created a platform to think of the possibility of developing rice rich in pro-vitamin A, known as Golden Rice [6]. The biochemical assay of the immature rice endosperm tissue with radiolabelled isoprenoid precursors indicated the presence of geranyl geranyl pyrophosphate (GGPP) in rice endosperm, which is the initial precursor molecule that gets converted into β-carotene, α-carotene and their derivatives [7]. However, the GGPP is not further converted to the downstream products *viz*, phytoene, lycopene, α and β-carotenes, indicating transcriptional inactivity of the genes governing the enzymes that catalyze the synthesis of these isoprenoid compounds in rice endosperm. Taking this cue, a transgenic approach was adopted to connect the missing links in β-carotene biosynthesis pathway. The proof of concept lines developed in the background of a *japonica* cultivar Taipei309, harbor the trans-genes encoding phytoene synthase (*Psy*) and lycopene cyclase (*Lcy*) genes from daffodil (*Narcissus pseudonarcissus*) and carotene desatuarase (*CrtI*) from the bacterium *Erwinia uredevora*. The β-carotene content of these prototype lines is as low as 1.7 μg/g of endosperm [4]. Further, the Golden Rice 1(GR1) series of lines developed in the background of an American long grain rice variety Cocodrie by Syngenta, accumulated carotenoids up to 7μg/g of endosperm [8]. But the highest increase in carotenoids levels was accomplished when the daffodil *PSY* was replaced by maize (*Zea mays*) enzyme with a much higher activity. Overexpression of the monocot *Psy* together with the bacterial desaturase *CrtI* in the rice endosperm was shown to boost the production of carotenoids, reaching up to 37 μg/g of dry weight [9]. The events developed using *ZmPsy* and *CrtI* both driven by endosperm-specific glutelin 1(Gt1) promoter from rice in the background of another American long grain rice variety Kaybonnet, were referred as Golden Rice2 (GR2) series. Six events (G1, R1, L1, T1, W1, and E1) of GR2 were made available for use in public sector breeding programs by Humanitarian Board (HumBo) on Golden Rice.

We, at the ICAR-Indian Agricultural Research Institute (ICAR-IARI), introgressed provitamin A trait from GR2-R1 event in to the background of mega rice variety, Swarna, adopting marker assisted backcross breeding to hasten the breeding cycle and develop near isogenic lines. Characterization of transgenic events in terms of the copy number of the transgenes, their stable Mendelian inheritance, their site of integration in the genome, site specific expression, position effect of the transgene and the effect of transgenesis on the overall phenotype of the donor line, other than the trait targeted, is prerequisite for further utilization of the transgenic germplasm in the downstream breeding programmes. A comparative study on the performance of transgenic lines and corresponding wild type and the near-isogenic lines (NILs) carrying transgenes developed through backcross breeding with recurrent parent, is crucial to see if there is any unintended effect of transgene insertion/introgression. Here, we report the morpho-molecular characterization of NILs of Swarna, which were developed using Kaybonnet-GR2-R1 event, containing transgene on chromosome 1 at physical location 38.75Mb [10].

## Material and methods

### Experimental material and growth conditions

Six transgenic lines developed in Kaybonnet background [9] and their respective null lines were obtained from Syngenta through HumBo under license agreement with Department of Biotechnology, Government of India. These events carried carotenogenic transgenes, *ZmPsy* from maize and *CrtI* from the bacterium *Erwinia uredevora*, both driven by endosperm-specific glutelin 1 (Gt1) promoter of rice, while the selectable marker gene phosphomannose isomerase (*pmi*) was driven by maize polyubiquitin (Ubi1) promoter. GR2-R1 event was used as donor to transfer the transgene into the genetic background of a mega rice variety ‘Swarna’, through marker assisted backcross breeding. The F_1_ developed by crossing Swarna as a female parent to the transgenic donor parent Kaybonnet, was backcrossed to Swarna to produce BC_1_F_1_ seeds. Seventeen BC_1_F_1_ plants were subjected to foreground selection by employing event specific PCR primers to identify transgene positive plants. Transgene positive plants were back crossed to Swarna to produce BC_2_F_1_ seed, BC_2_F_1_ population, followed by foreground analysis and the identified transgene positive plants were subjected to background selection with eight STMS markers on the carrier chromosome and 61 polymorphic markers between donor and recurrent parents uniformly distributed in the remaining 11 chromosomes. Background analysis recovered more than 96% of the recurrent parent genome (RPG) in just two backcrosses. Three plants with maximum recovery of RPG were selected for generation advancement. The material for the present study consisted of BC_4_F_2_ plants, which were grown under containment in the Transgenic Screen House at the Division of Genetics, ICAR-IARI, New Delhi, following the recommended package of guidelines and practices. In preliminary analysis, it was observed that the lines homozygous for transgene were found to have drastically altered phenotype as compared to their hemizygous and null siblings and therefore an in depth analysis was planned to understand its morpho-molecular basis.

### Zygosity determination of backcross derived lines

Leaf samples were collected from seedlings at 15 days after transplanting. The genomic DNA was extracted as described previously [11]. An event specific three polymerase chain reaction (PCR) primers (personal communication, HumBo) capable of differentiating homozygous, hemizygous and null plants were used to identify the zygosity of plants within progeny. On PCR amplification, the transgene homozygous and null plants were characterized by a single fragment of 734 bp and 449 bp, respectively, while the hemizygous plants amplified both these fragments. The PCR reactions were carried out using sterile 96-well PCR plates obtained from Axygen Scientific Inc. (Union city, CA, USA). The master mix consisted of 25 ng of genomic DNA, 0.6 U of *Taq* DNA polymerase, 1X PCR assay buffer without MgCl_2_, 1.75 mM MgCl_2_, 200 μM of dNTP mix and three primers at a concentration of 1 μM each. The reaction volume was made up to 20 μl using sterile double distilled water. The amplified products were resolved on 1.2% agarose gel stained with ethidium bromide (10 mg/ml). The gel was run in 1X TAE buffer (pH 8.0). DNA fragments were visualized under UV light and the image was documented in ultraviolet transilluminator (Gel Doc^TM^ XR+ Imager, Bio-Rad Laboratories Inc., U.S.A). Based on the PCR analysis, ten plants each of homozygous, hemizygous and null lines were tagged in the screen house and subsequently, observations were recorded in these plants.

### Phenotypic characterization of backcross derived golden rice lines

At maturity, the homozygous, hemizygous and null plants were characterized in terms of various traits of agronomic importance *viz*. plant height, heading date, panicle length, percent panicle exertion, flag leaf length, tiller number, effective tiller number, spikelet number per panicle, percent spikelet fertility and 1000 grain weight. In addition, the germination behavior of these lines was also monitored by conducting germination tests on petri plates with blotting paper saturated with water. Roots of 20 days old seedlings grown in hydroponics were scanned and analyzed in WinRHIZO software (Regent Instruments, Inc., Quebec, QC, Canada)

### Quantification of phytohormones

Samples (leaf blade, leaf sheath, panicle, and internode) collected at booting stage in two biological replicates were analyzed for the level of different phytohormones: gibberellin A_3_ (GA_3_), indole-3-acetic acid (IAA), trans-zeatin (tZ) and abscisic acid (ABA). The samples after harvesting, were immediately frozen in liquid N_2_ and stored at -80°C until further analysis. The phytohormone extraction was done according to the method described previously [12], with slight modifications. The tissue was ground to fine powder in liquid N_2_ and 10 g of powder was used for extraction with 70% (v/v) methanol as a solvent. The samples were incubated overnight at 4°C under continuous gentle stirring. After filtration, the residue was re-extracted twice with methanol and refiltered. The methanol portion of the combined extracts was evaporated using rotary evaporator at about 35°C. The flask used for rotary evaporation was rinsed with 5 ml of water. The pH of the aqueous portion was adjusted to 8.5 using NaOH and partitioned thrice with ethyl acetate. The ethyl acetate portion containing chlorophyll pigments was discarded and the pH of the aqueous portion was again adjusted to 2.5 with 1N HCl and partitioned thrice with diethyl ether. The aqueous portion was discarded and ether portion was collected and passed through anhydrous Na_2_CO_3_ to remove any traces of water. Diethyl ether was completely dried and finally dissolved in 1 ml of 100% methanol and the final aliquot was stored in a vial at 4°C.

The phytohormones in the extract were quantified by High Performance Liquid Chromatography (HPLC) (Waters Ltd., Elstree, Herts, UK) using a reverse phase C18 column (150 mm x 4.6 mm i.d; 5 μm) maintained at a constant temperature of 30 ± 1°C. After passing the aliquot through a 0.22 mm Millipore filter, 10 μl was injected into the column for analysis. The mobile phase consisted of methanol and water; the pH of both was adjusted to 6.8 using dilute acetic acid. A flow rate of 0.75 ml/min was maintained and the mobile phase was delivered in a gradient mode with 30% of A for initial 5 min, 50% of A from next 5–15 min, 100% of A from 15–20 min, and 30% A for 20–25 min. The wavelengths of 208, 247, and 268 nm were used for the detection of GA, ABA, and tZ, respectively, in a photodiode array detector while IAA was monitored in a fluorescence detector under a wavelength of 254 nm for excitation and 360 nm for emission. Different concentrations of the chemical-grade standards (obtained from Sigma Aldrich) prepared in 100% methanol were injected to determine the retention time. The peak areas were measured and a calibration curve was constructed for each compound by plotting the area under the peak against the concentration. The phytohormone concentration in the sample was determined by extrapolating its area in the standard curve.

### Quantification of chlorophyll

The amount of chlorophyll present in the samples was estimated using DMSO technique [13]. Leaf discs weighing 50 mg were put into the glass centrifuge vials containing 5 ml DMSO preheated to 65°C in a water bath. When the extractions were complete, samples were removed from the water bath and each graduated vial was topped up to exactly 10 ml with DMSO using a Pasteur pipette; 1 ml of each extract were then transferred to glass cuvettes with a reported standard deviation between cuvettes of < ±0.005 extinction units. The spectrophotometer was calibrated to zero absorbance using a blank of pure DMSO. Absorbance of both blank and samples were measured at 645 and 663 nm. The chlorophyll concentration was then calculated using Arnon’s [14] equations: Chla (g l−1) = 0.0127 A663–0.00269 A645; Chlb (g l−1) = 0.0229 A645–0.00468 A663; total Chl (g l−1) = 0.0202 A645 + 0.00802 A663.

### *In silico* analysis

To ascertain if transgene insertion has disrupted any native gene, the genomic region of chromosome 1 containing the transgene was scanned by using the flanking event specific PCR primers provided by Syngenta through internal communication, for zygosity detection, as query sequences against Nipponbare rice genome as reference using NCBI nucleotide blast tool (http://blast.ncbi.nlm.nih.gov/Blast.cgi).

### Gene expression analysis

Total RNA was extracted from ~100 mg of different tissues (leaf blade, leaf sheath, panicle, root and internode) from null, hemizygous and homozygous plants using RNeasy Plant minikit (Qiagen, Germany) using manufacturer’s protocol. Thereafter, first-strand cDNA was synthesized from *DNase* I-treated total RNA using Transcriptor First Strand cDNA synthesis kit (Roche, Germany) in a thermal cycler (Veriti Thermal Cycler, Applied Biosystems, USA) according to manufacturer’s instructions. The primers for real-time PCR was made preferably from 3’UTR of the respective genes using Primer Express 2.0 software (PE Applied Biosystems, USA); the specificity of each primer set was checked by performing BLAST search in RGAP (Rice Genome Annotation Project) database. The real-time PCR reaction mixture containing cDNA as template, gene-specific primers and SYBR green PCR Mastermix (Roche, Germany) was prepared in dim light. The real-time PCR was performed in 96-well plate of LightCycler 480 II real-time machine (Roche, Germany), using the following conditions: initial denaturation at 95°C for 10 min, followed by 45 cycles of denaturation at 95°C for 10 s, primer annealing at 60°C for 20 s, and extension at 72°C for 10 s. The specificity of PCR products was verified by performing melting curve analysis at 95°C for 5 s, 65°C for 1 min and continuous 97° C with acquisition per 5°C. Rice *ubiquitin 5* gene was used as an internal control for normalization, to ensure equal amount of RNA was taken for each sample. The Ct method [15] was applied to calculate the relative amount of target gene expression, which is presented as the quantity normalized to the endogenous reference *ubiquitin 5* and relative to the calibrator. For each gene, at least two biological replicates, each having three technical replicates was taken for analysis. The gene specific primer sequences used for the expression analysis are given in S1 Table.

### Statistical analysis

For comparisons among the three groups of genotypes (null, hemizygous, homozygous), one way ANOVA was carried out using SPSS software, and Tukey’s test was adopted as a post hoc test to identify the groups that are significantly different from the other for all the agro-morphological traits.

## Results

### Abnormal phenotype of the homozygous plants

**Fig 1:**
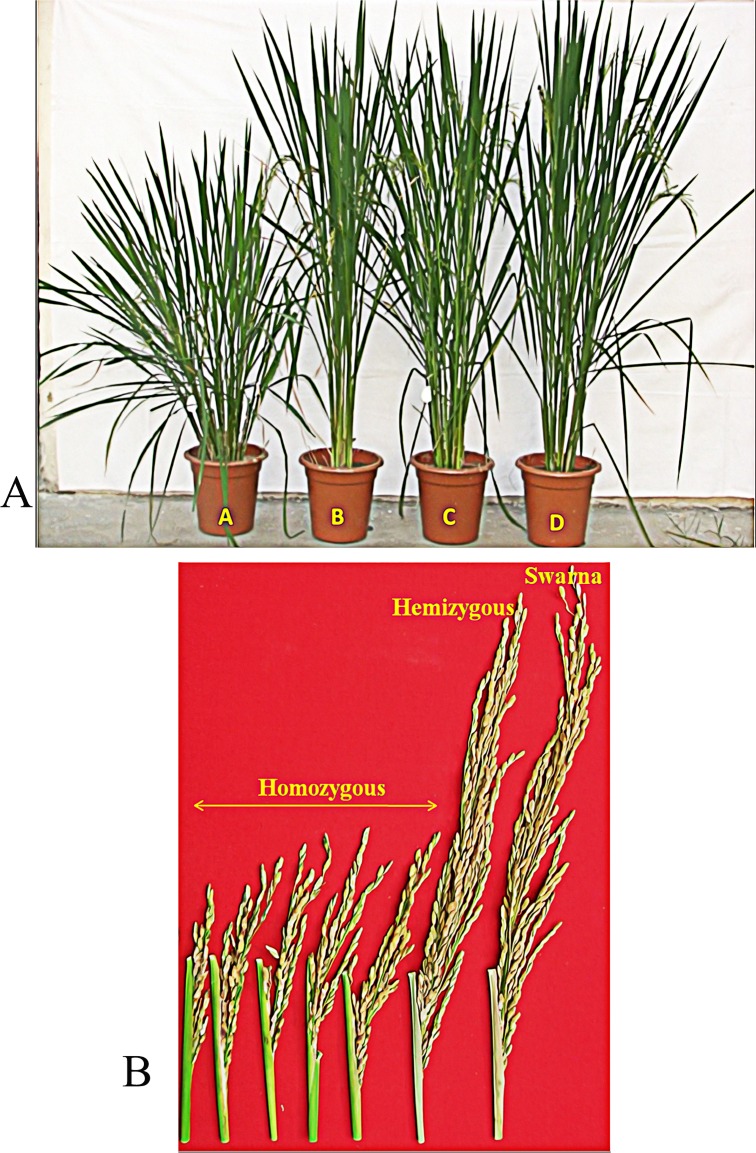
**Comparison of plant phenotype among transgene homozygous (A), hemizygous (B) and null (C) lines in relation to the RP Swarna (D).** A) Reduced plant height of transgene homozygous plant in comparison to a hemizygous plant and the RP Swarna. B) Reduced panicle size and poor panicle exertion in a transgene homozygous plant in comparison to a hemizygous plant and the RP Swarna.

The homozygous, hemizygous and null plants were identified based on the event specific PCR analysis, as described in the material and methods. A representative gel picture depicting different genotypes is given in S1 Fig. The plants homozygous for the transgene cassette were shorter in height (47.2 ± 5.5 cm, n = 10) as compared to the hemizygous (87.2 ± 10.6 cm, n = 10) and null (91 ± 6.8 cm, n = 10) plants (Fig 1A). In addition, these plants exhibited delayed heading (by nine days), incomplete panicle emergence (46.2% of the panicle remaining enclosed in the leaf sheath) and smaller panicle size (13.9 ± 3.2 cm) than hemizygous (26.2 ± 2.2 cm) and null (25.3 ± 2.7 cm) plants (Fig 1B). Moreover, these homozygous transgenic plants displayed two times more tillers when compared to the hemizygous and null plants. The homozygous transgenic plants also produced atypical nodal branching in the first node above the ground. However, there was significant reduction in spikelet fertility of homozygous plants compared to the hemizygous and null siblings (S2 Table). The thousand seed weight of homozygous plants was on par with that of hemizygous and nulls. There was a drastic reduction in the yield of homozygous plants (8.3 g ± 2.5) compared to those of nulls (23.24 g ± 2.4) Similar observations were also made in the transgenic Kaybonnet plants (containing the transgene cassette) which displayed reduced plant height, increased leaf number, pale green leaves, reduced flag leaf length, increased tiller number, reduced panicle size and incomplete panicle exertion when compared to its null (S2 Fig).

### Alteration in germination behavior and root phenotype of homozygous lines

**Fig 2:**
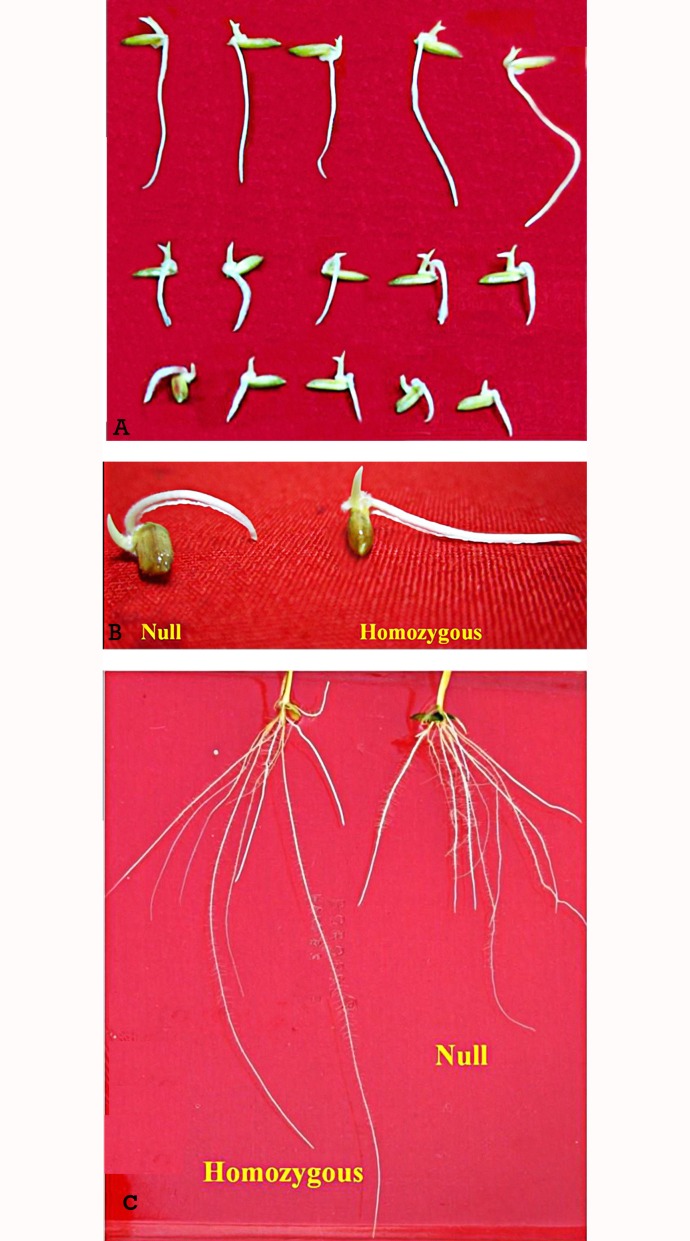
Characterization of root phenotype and germination behaviour of backcross derived Golden Rice lines. A) Comparison of primary root length among the seedlings of transgene homozygous, null and Swarna The embryonic axis showed rapid elongation in transgene homozygous seedlings resulting in longer primary root. The picture was taken at three days post germination. B) Orientation of the primary root in the transgene homozygous and null seedlings upon germination A typical curvature pointing towards gravity as seen in the Null seedling was absent in the transgene homozygous seedling. The picture was taken at two days post germination C) Comparative view of root system in the transgene homozygous and null plants The seedlings were grown in hydroponics with Hoagland nutrient solution and the roots of 20 days old seedlings were analyzed in Root Scanner device. The total root length and root volume of homozygous plants was significantly higher than those of nulls D) ABA insensitive germination of the transgene homozygous seeds The upper panel shows the germination under control (water) and the bottom panel shows the germination under the exogenous supply of ABA (1ppm). ABA repressed the germination in nulls (bottom left) while it does not have any effect on the germination of homozygous seeds (bottom right).

Germination tests revealed that the homozygous lines do not differ from the nulls for the time of seed germination; they, however, show rapid elongation of the embryonic axis (primary root), so that at any given point of time, the embryonic axis of transgene homozygous plants is significantly longer than that of nulls (Fig 2A). Moreover, the primary root of homozygous plants also differs in its orientation; the primary root of a null line shows a typical curvature pointing towards the gravity, while this curvature is absent in that of homozygous plants (Fig 2B). Further, the total root length and root volume of homozygous plants was significantly greater (969.61 cm and 1.64 cm^3^, respectively) than the corresponding null plants (607.4 cm and 0.65 cm^3^ respectively) when grown hydroponically for one month, as analyzed by the root scanner device (Fig 2C).

Furthermore, the transgene homozygous seeds germinated in the presence of 100 μM ABA, failed to show repression, while the germination was repressed in the null lines under same condition (Fig 2D).

### Imbalance in hormonal homeostasis

**Fig 3:**
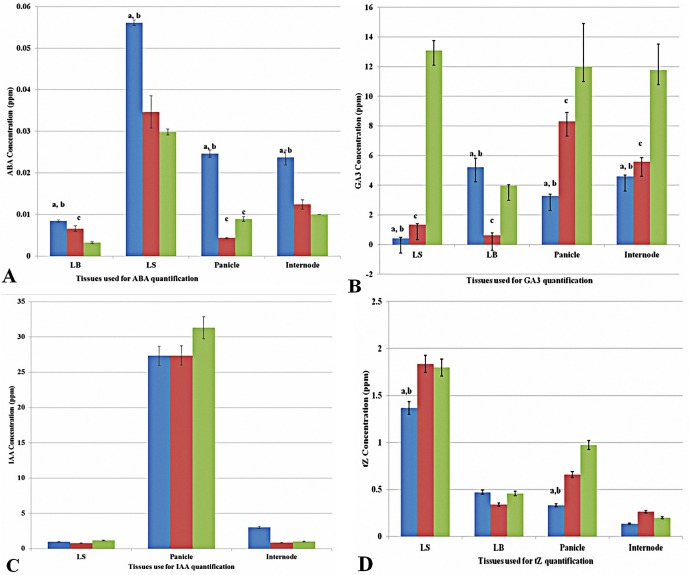
Alteration in hormonal homeostasis of transgene homozygous lines in relation to the hemizygous and null lines. A) Relative concentration of ABA in transgene homozygous, hemizygous and null lines, analyzed in leaf blades (LB), Leaf sheath (LS), panicle and internodal tissues. B) Relative concentration of GA3 in transgene homozygous, hemizygous and null lines analyzed in leaf blades (LB), Leaf sheath (LS), panicle and internodal tissues. C) Relative concentration of IAA in homozygous, hemizygous and null lines analyzed in Leaf sheath (LS), panicle and internodal tissues. D) Relative concentration of tZ in transgene homozygous, hemizygous and null lines analyzed in leaf blades (LB), Leaf sheath (LS), panicle and internodal tissues. The error bars represent SE, n = 2. a refers to significant difference of homozygous from hemizygous, b significant difference of homozygous from null and c significant difference of hemizygous from null at P = 0.05 significance level.

To elucidate the biochemical basis of these morphological changes, phytohormone concentrations were measured in four different tissues *viz*. leaf sheath (LS), leaf blade (LB), internode (I) and panicle (P). The concentration of ABA was found to be significantly higher in the homozygous plants in all the tissues analyzed. Except for the LB, where hemizygous plants showed significantly higher concentration than the nulls, in all the other tissues similar concentration was found in the hemizygous and null plants (Fig 3A). Homozygous plants had much lower GA_3_ levels in the LS, I and P tissues whereas in the LB tissue, higher concentration was recorded (Fig 3B). The concentration of IAA was not found to be significantly different among the plants differing for their zygosity of the transgene, in all tissues analyzed (Fig 3C). The concentration of tZ was found to be lower in the LS, P and I tissues of homozygous plants (Fig 3D).

### Reduction in chlorophyll pigments

The total chlorophyll content of homozygous plants was significantly lower than hemizygous and null plants, with reduction occurring in both chlorophyll a (Chla) and chlorophyll b (Chlb) levels. (S3 Fig). Hemizygous plants contain similar levels of Chla, but significantly lower Chlb levels than the null plants.

### *In silico* analysis reveals the disruption of native *OsAux1* gene

**Fig 4:**
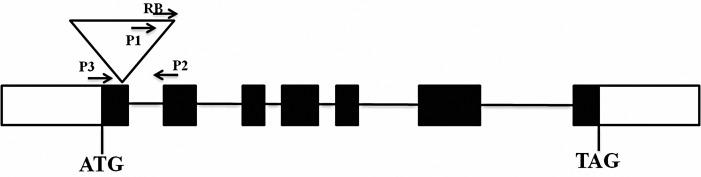
Schematic diagram showing the position of transgene integration in exon 1 of *OsAux1* (*Os01g0856500*) gene at 151bp position. The gene codes for auxin influx carrier protein which is a trans-membrane transporter that regulates the flow of auxin in to the cell. The size of OsAux1 gene is 6.3Kb and transgene integration occurred after 151th bp of exon 1. P1, P2 and P3 represent the positions of event specific PCR primers.

Syngenta (through personal communication) supplied a protocol for zygosity detection through event specific PCR analysis using a three primer combination of which the primer 1 was from transgene sequence and primers 2 and 3 were from the flanking rice genomic sequences. When these primers were queried against Nipponbare reference sequence in BLAST analysis, primer 2 and primer 3 showed hits in exon 1 and exon 2 respectively, of *OsAux1* gene (*Os01g0856500*), located on chromosome 1, indicating that the transgenic construct for the expression of provitamin A trait was inserted somewhere between these two exons (Fig 4). As the event-specific PCR using three primer combination amplifies a 734 bp product of which 412 bp is from the transgene construct, the remaining 322 bp should be from the sequence of the *OsAux1* gene as the primers 2 and 3 showed complementarities on the exon 1 and 2 of the gene. Sequence analysis further revealed that this 322bp flanking sequence includes140bp (including primer 3 sequence) from the exon 2, 132bp from the intron 1 and the remaining 50bp (including primer 2 sequence) from exon 1. This clearly demonstrates that the transgene insertion has occurred in the exon 1 of *OsAux1*, disrupting its coding sequence and affecting its function.

### Negligible expression of *OsAux1* in transgene homozygous lines

**Fig 5:**
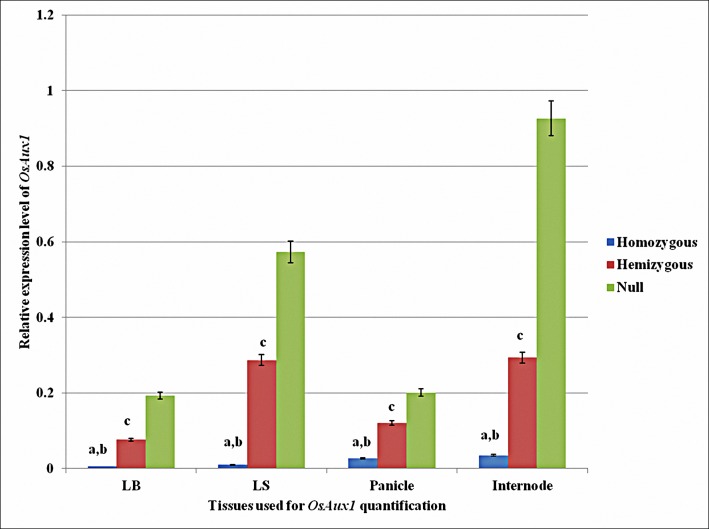
Relative concentration of *OsAux1* gene in leaf blade (LB), leaf sheath (LS), panicle and inter-nodal tissues of transgene homozygous, hemizygous and null plants. The transcripts of the gene were totally absent in the transgene homozygous plants in all the tissues analyzed while hemizygous and null plants differ significantly in their expression levels. The error bars represent SE, n = 3. a refers to significant difference of homozygous from hemizygous, b significant difference of homozygous from null and c significant difference of hemizygous from null at P = 0.05 significance level.

The expression level of *OsAux1* gene quantified in homozygous, hemizygous and null siblings derived from a single BC_4_F_1_ hemizygous plant using real time PCR analysis showed that the *OsAux1* transcripts were negligible in the homozygous plants in which both the copies of the *OsAux1* gene were disrupted as a result of transgene integration. In the hemizygous lines with one functional copy of the *OsAux1* gene, the expression was approximately half as compared to null lines with both functional copies of the *OsAux1* gene (Fig 5). This variation in expression level of *OsAux1* transcript in the homozygous and hemizygous lines indicates that the transgene insertion has resulted in loss of function of *OsAux1* gene in the transgenic lines there by affecting the plant morphology and agronomic performance of SGR2-R1 event derived lines.

### Non-specific expression of endosperm specific Gt1 promoter

**Fig 6:**
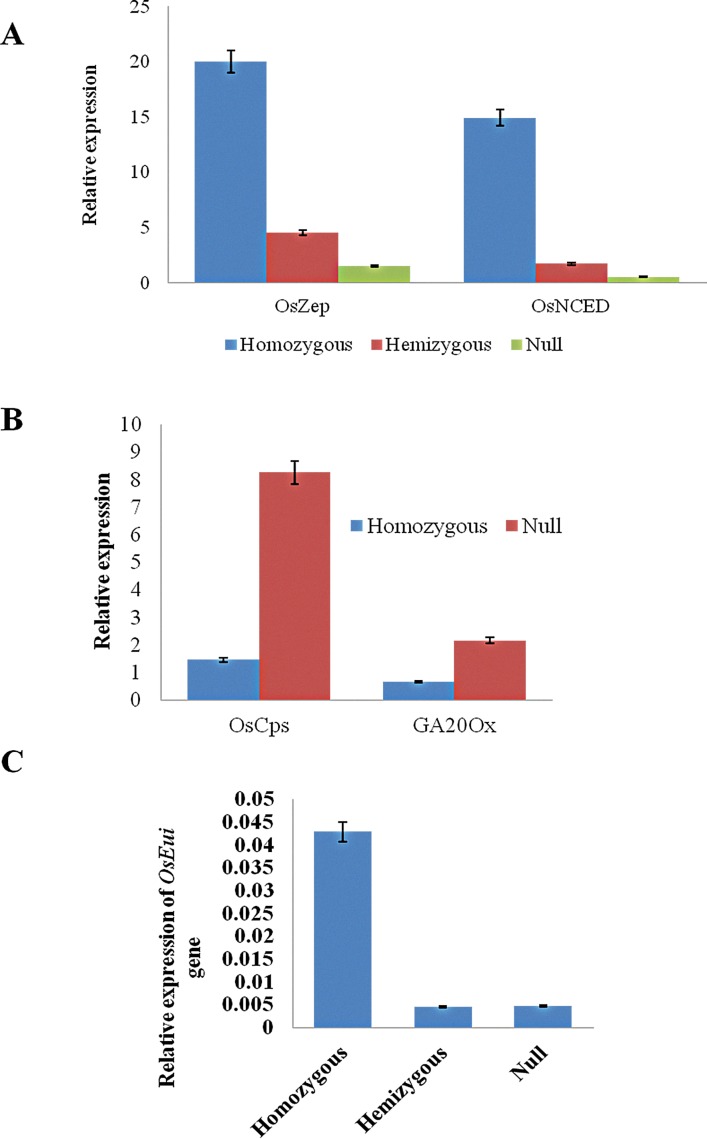
**Molecular characterization of backcross derived Golden Rice lines.** A) Relative expression of Os*Zep* and OsNCED genes involved in ABA biosynthesis, in the leaf tissues of transgene homozygous, hemizygous and null plants. B) Relative expression of *OsCps* and GA20ox genes involved in GA biosynthesis, in leaf tissues of transgene homozygous and hemizygous plants. C) Relative expression of *OsEui* gene involved in GA deactivation, in the panicles of transgene homozygous and hemizygous and null plants. Error bars represent SE, n = 3.

The expression analysis of two transgenes, *ZmPsy* and *CrtI*, revealed the presence of transcripts in vegetative tissues of leaf blade (LB). This was an unexpected observation since both the genes are driven by an endosperm-specific Gt1 promoter, and hence should not express in tissues outside the endosperm. The expression of these genes was two-fold higher in homozygous lines compared to the hemizygous ones (S4 and S5 Figs), while the expression was absent in null lines. We hypothesize that the leaky expression of these transgenes would have altered the expression level of genes involved in GA biosynthetic pathway since the carotenoid biosynthetic pathway diverges from the same substrate, geranyl-geranyl bi-phosphate (GGPP). Furthermore, β-carotene is metabolized to another growth regulator, ABA, which is antagonistic to GA. Therefore, enhanced expression of β-carotene is also likely to alter ABA expression. This hypothesis was tested by quantifying the expression levels of four key genes two each in ABA and GA biosynthesis pathways. Real time PCR analysis of *OsZep* gene encoding zeaxanthin epoxidase, which catalyses the first step in the production of ABA, *i*.*e*. the conversion of zeaxanthin to violaxanthin, revealed that the expression of this gene was 20-fold higher in transgene homozygous plants and ~4-fold higher in hemizygous plants compared to the nulls while the expression of *OsNCED* gene, which codes for 9-cis epoxycarotenoid dioxygenase that cleaves 9-cis xanthophylls to xanthoxin, a precursor of ABA was found to be 15 folds higher in homozygous plants and 3 folds higher in hemizygous plants when compared to the nulls (Fig 6A). Expression analysis of key genes in GA biosynthesis pathway revealed that the gene *OsCps* encoding ent-copalyl phosphate synthase, which catalyzes the conversion GGPP to ent-copalyl diphosphate in the plastids, was found to be 6-fold lower in the transgene homozygous plants compared to their null siblings while the expression of GA20oxidase gene was found to be 3.3 fold higher in homozygous plants than their null siblings (Fig 6B). Furthermore, the expression of Elongated Uppermost Internode (EUI) gene which is involved in GA catabolism, was found to be higher in transgene homozygous plants compared to hemizygous and null plants (Fig 6C).

## Discussion

Introgression of the provitamin A trait from *javanica* rice variety ‘Kaybonnet’, into the background of popular high yielding *indica* rice cultivars is crucial to address the problem of VAD in India. MABB was adopted to transfer the carotenogenic transgenes into the background of mega rice variety ‘Swarna’. The backcross derived Golden Rice lines of Swarna were characterized for their morphological traits and agronomic performance, which revealed that the line homozygous for transgene had several aberrations in their phenotype. One of the major factors affecting the successful adoption of the improved lines is their relative performance as compared to the recurrent parent for yield. The present study revealed significant alteration in the hormonal homeostasis of the homozygous plants due to the disruption of endogenous *OsAux1* gene by the transgene insertion.

The ideal transgenic plant for most research and breeding purposes would contain a single intact copy of the desired transgene inserted into the host genome, such that it does not alter functionality of the host plant DNA [16]. *Agrobacterium*-mediated transformation leads to random insertion of the transgenes in the genome. Therefore, it is very important to determine the genomic location of the transgene insertion. Data accumulated from several large-scale T-DNA tagging experiments in both *Arabidopsis thaliana* and rice suggest that with *Agrobacterium*-mediated transformation, T-DNA insertion into genic sequences ranges from 35–58% of T-DNA insertion events [17–21]. In the present study, we observed disruption of a native *OsAux1* gene as a result of the insertion of carotenogenic transgene in GR2-R1 event through *in silico* analysis of the flanking sequences. Real time PCR analysis confirmed the disruption of *OsAux1* in transgene homozygous plants where there was complete absence of the *OsAux1* transcripts. The endogenous *Aux1* gene codes for an auxin influx carrier protein. *Aux1* belonging to the amino acid/auxin permease family, mediates the distribution of IAA between sink and source tissues in *Arabidopsis* [22–25]. *AtAUX1* regulates root gravitropic curvature and promotes lateral root formation by acting in unison with the auxin efflux carriers to co-ordinate the localized redistribution of auxin within the *Arabidopsis* root apex [26–29]. Mutations within the *Aux1* gene confer an auxin-insensitive phenotype. Reduced rate of carrier-mediated auxin uptake in the *aux1* mutants confers an agravitropic root phenotype with decreased lateral roots in *Arabidopsis* [30, 31]. Rice genome encodes five *Aux-*like genes named as *OsAux1-5*, with *OsAux1* sharing highest similarity (82.89%) with *AtAux1* [32]. In the present study, we report the absence of gravitropic curvature due to disruption of *Aux1* in the transgene homozygous plants. The primary root growth and the root phenotype of transgene homozygous plants resemble that of *aux1* mutants of *Arabidopsis*. Further, the atypical nodal branching in the transgene homozygous plants clearly indicates the reduced apical dominance, confirming the disturbance in auxin homeostasis.

The inhibitory action of ABA on embryonic axis elongation and cotyledon expansion during seed germination and early seedling growth is well established [33–36]. However, we observed that the embryonic axis of the transgene homozygous seeds did not show any repression upon exogenous supply of ABA, while in the corresponding null seeds, the growth of the embryonic axis was repressed. This observation was confirmed in the transgenic donor ‘Kaybonnet’ and their nulls as well. In *Arabidopsis* also, AUX1 has been shown to be involved in ABA-dependent repression of embryonic axis elongation during early seedling development [37]. The faster axis elongation in *aux1* embryos in the absence of exogenous ABA may reflect their resistance to the endogenous ABA pools present in germinating seeds [38]. This along with the findings in the present study demonstrates the conserved role of AUX1 in *Arabidopsis* and Rice.

Furthermore, the leaky expression of endosperm-specific Gt1 promoter in the vegetative tissues as observed in the backcross derived transgenic Golden Rice lines in ‘Swarna’ possibly lead to the competition among the pathways dependent on GGPP as a substrate, as the pathways for β-carotene, GA, ABA and chlorophyll biosynthesis are interconnected through the common substrate GGPP [39, 40]. Any change in one pathway would influence the others as well [41, 42]. In the transgene homozygous lines, endogenous rice *Psy* together with the transgene *ZmPsy*, lead to the over production of PSY enzyme which may have converted GGPP to phytoene and thereby diverting this intermediate from the GA biosynthesis and other phytol biosynthesis pathways towards β- carotene pathway. Furthermore, the downstream of β-carotene biosynthesis pathway leads to the production of ABA, which is antagonistic to GA [43, 44]. In *Arabidopsis*, a high endogenous level of ABA causes a reduction in the endogenous level of GA [45, 46].

A novel mechanism of GA deactivation was demonstrated [47, 48] in rice that is governed by *Eui* gene, which encodes a cytochrome P450 monooxygenase that catalyzes 16α,17-epoxidation of non-13-hydroxylated GAs to generate bio-inactive 16α,17-[OH]2-GAs. Therefore, overexpression of EUI causes a dwarf phenotype, whereas mutation within this gene increases the internode length in rice. Furthermore, Yaish et al. demonstrated that the presence of ABA Response Element motif (CACGTG) in the EUI promoter at -2355 bp from the ATG start codon, and showed that endogenous or exogenous ABA induces the expression of EUI which in turn would lead to GA deactivation [49]. Our biochemical analysis revealed the presence of higher levels of ABA and lower levels of GA_3_ in all the tissues analyzed and the gene expression analysis revealed the higher level of expression of *Eui* gene in the transgene homozygous lines. This suggests that low GA levels in transgene homozygous lines can be attributed to the antagonistic effect of high ABA levels mediated through higher expression of *OsEUI* gene. The substrate competition between GA and β-carotene pathway diverts the flux of GGPP towards β-carotene pathway and leads to further reduction in the endogenous GA levels, which has been confirmed through the reduced expression of *OsCps* gene (regulating the first committed step in the GA biosynthesis pathway, converting GGPP to ent Kaurene) in the transgene homozygous lines.

The pale green leaves in homozygous plants are probably due to lower concentration of chlorophyll pigments, as a result of substrate competition, since GGPP is also the source of phytol tail of chlorophyll. It can also be explained in terms of auxin-induced reduction in the concentration of another phytohormone, cytokinin, which is known to be involved in chlorophyll biogenesis. Moreover, cytokinin deficient mutants show accelerated leaf senescence. It has been demonstrated that auxins controls cytokinin biosynthesis [50, 51] and most auxin-resistant mutants also show changes in their cytokinin sensitivity [52, 53].

Phytohormones regulate a plethora of processes in the life cycle of a plant, at various stages, starting from embryo development, seed germination, root growth, vascular differentiation and shoot architecture flower development and response to various biotic and abiotic stresses. The activity of every plant hormone is determined by its availability, which is controlled at the level of metabolism and distribution, and by the efficiency of the hormonal signal perception and transduction. Modulation of any of these might have a direct impact on downstream responses such as target gene expression or protein activity control. Furthermore, as the hormones work in a coordinated manner in maintaining the essential functions of the plants, any disturbance in the metabolism or transport of one hormone influences the metabolism of other hormones [54–59]. Our study clearly demonstrates, how the disruption of a single major gene, *OsAux1*, involved in the transport of master growth regulator auxin, results in an array of abnormalities in the germination behavior, root growth and shoot phenotype of the transgene homozygous plants. Coupled to this, the leaky expression of endosperm-specific promoter has also resulted in substrate competition among the GGPP dependent pathways. However, demonstrating the corresponding magnitude of these two phenomena is complicated owing to the complexity of interactions among various hormones. The increased tiller number and atypical nodal branching in the homozygous plants indicates reduced apical dominance in transgene homozygous plants that could be attributed to the disturbance in auxin distribution caused by disruption of *OsAux1*. The root phenotype observed in this study correlates well with that of *aux1* mutants in *Arabidopsis*. Auxin is considered as a looping star, virtually involved in every aspect of plant growth and development [60, 61]. It is known to influence GA biosynthesis by up-regulating the expression of GA biosynthesis genes *viz*. *GA20oxidase* and *GA3oxidase* [62–66]. Our study in rice and previous reports in *Arabidopsis* shows that any disturbance in auxin balance could affect the balance of other hormones.

## Conclusion

Based on the morphological, biochemical and molecular characterization of backcross derived transgenic Golden Rice lines in the genetic background of Swarna, we discovered that the insertion of transgene for pro-vitamin A trait in the donor GR2-R1 event in Kaybonnet, had disrupted a native *OsAux1* gene, which resulted in phenotypic abnormality and poor agronomic performance of the backcross derived Golden Swarna lines, making them unfit for commercial cultivation inspite of having high provitamin A content. The study conclusively demonstrates the importance of event characterization and event selection before adopting the transgenic germplasm into introgression breeding.

## Supporting Information

S1 Fig A representative gel picture depicting the genotypes of Swarna (S), Kaybonnet (K), transgene Homozygous (1), Hemizygous (2) and Null (3).
(PDF)

S2 Fig (**A**)Comparison of plant height between the transgenic donor parent Kaybonnet and its null (**B)** Comparison of panicle size between the transgenic donor parent Kaybonnet and its null.
(PDF)

S3 Fig Relative concentration of chlorophyll pigments in the flag leaf tissue of the three groups of genotypes. The error bars represent SE, n = 3. a refers to significant difference of homozygous from hemizygous, b significant difference of homozygous from null and c significant difference of hemizygous from null at P = 0.05 significance level.
(PDF)

S4 Fig Relative expression of *ZmPsy* in the leaf blades of transgene homozygous, hemizygous and null lines. Primers were designed complementary to the *ZmPsy* sequence of the transgene. These primers when blasted to rice genome failed to show any hits and this indicate that the observed transcripts are not due to the amplification of endogenous rice *Psy*. The error bars represent SE, n = 3.
(PDF)

S5 Fig Relative expression of *CrtI* in the leaf blades of transgene homozygous, hemizygous and null lines. The error bars represent SE, n = 3.
(PDF)

S1 Table Primer sequences used for expression analysis. (DOCX)

S2 Table Agronomic performance of the backcross derived lines varying in their transgene zygosity in the background of mega rice variety Swarna. (DOCX)
