# Supplementary material for: Molecular and Functional Characterization of GR2-R1 Event Based Backcross Derived Lines of Golden Rice in the Genetic Background of a Mega Rice Variety Swarna
Source: PLoS One. 2017 Jan 9;12(1):e0169600. doi: 10.1371/journal.pone.0169600 (PMC5221763; doi:10.1371/journal.pone.0169600)
Supplement: S1 Fig — A representative gel picture depicting the genotypes of Swarna (S), Kaybonnet (K), transgene Homozygous (1), Hemizygous (2) and Null (3). (PDF) [file pone.0169600.s001.pdf]

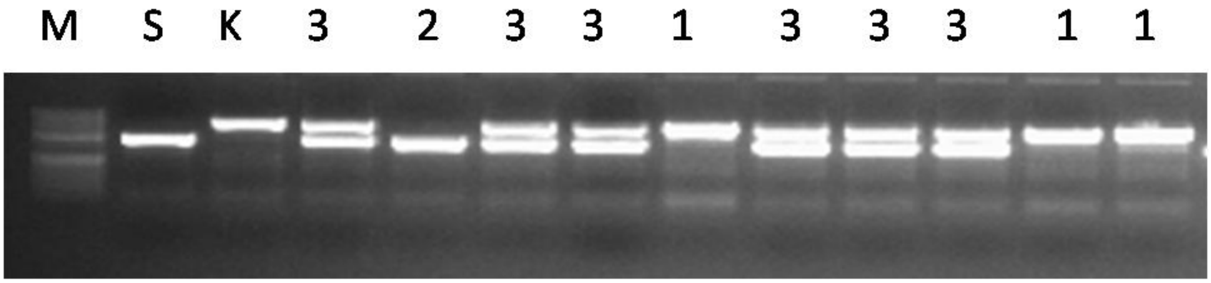

S1 Fig. A representative gel picture depicting the genotypes of Swarna (S), Kaybonnet (K), Homozygous (1), Null (2) and Hemizygous (3)
