# Supplementary material for: Molecular and Functional Characterization of GR2-R1 Event Based Backcross Derived Lines of Golden Rice in the Genetic Background of a Mega Rice Variety Swarna
Source: PLoS One. 2017 Jan 9;12(1):e0169600. doi: 10.1371/journal.pone.0169600 (PMC5221763; doi:10.1371/journal.pone.0169600)
Supplement: S2 Fig — (A)Comparison of plant height between the transgenic donor parent Kaybonnet and its null (B) Comparison of panicle size between the transgenic donor parent Kaybonnet and its null. (PDF) [file pone.0169600.s002.pdf]

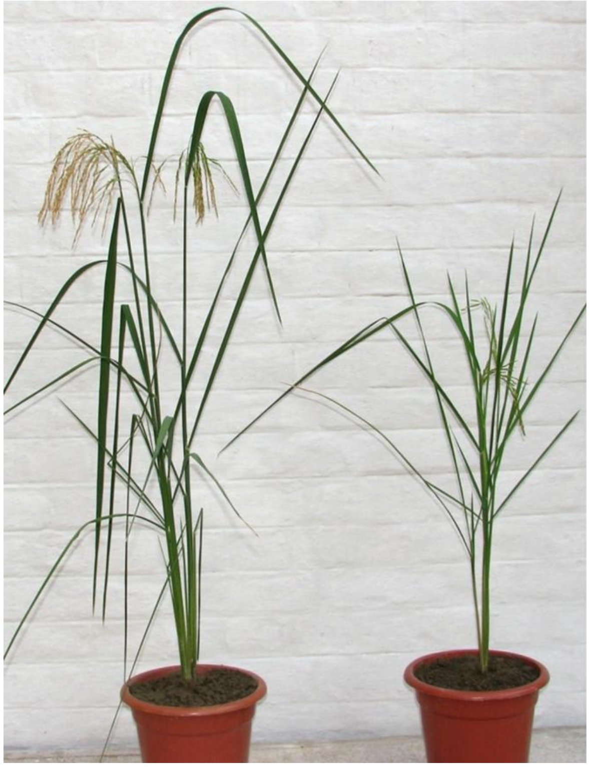

S2A Fig. Comparison of plant height between the transgenic donor parent Kaybonnet and its null

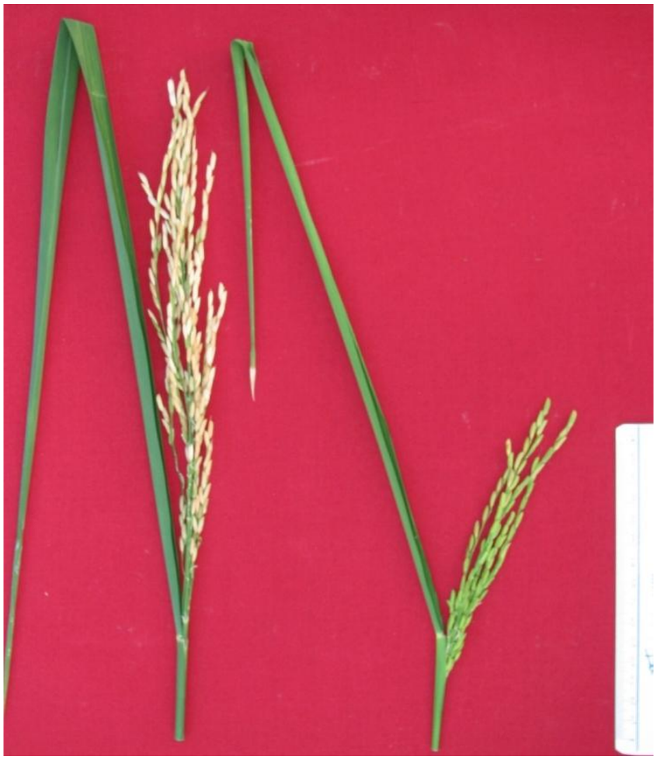

S2B Fig. Comparison of panicle size between the transgenic donor parent Kaybonnet and its null
