# Supplementary material for: Molecular and Functional Characterization of GR2-R1 Event Based Backcross Derived Lines of Golden Rice in the Genetic Background of a Mega Rice Variety Swarna
Source: PLoS One. 2017 Jan 9;12(1):e0169600. doi: 10.1371/journal.pone.0169600 (PMC5221763; doi:10.1371/journal.pone.0169600)
Supplement: S3 Fig — The error bars represent SE, n = 3. a refers to significant difference of homozygous from hemizygous, b significant difference of homozygous from null and c significant difference of hemizygous from null at P = 0.05 significance level. (PDF) [file pone.0169600.s003.pdf]

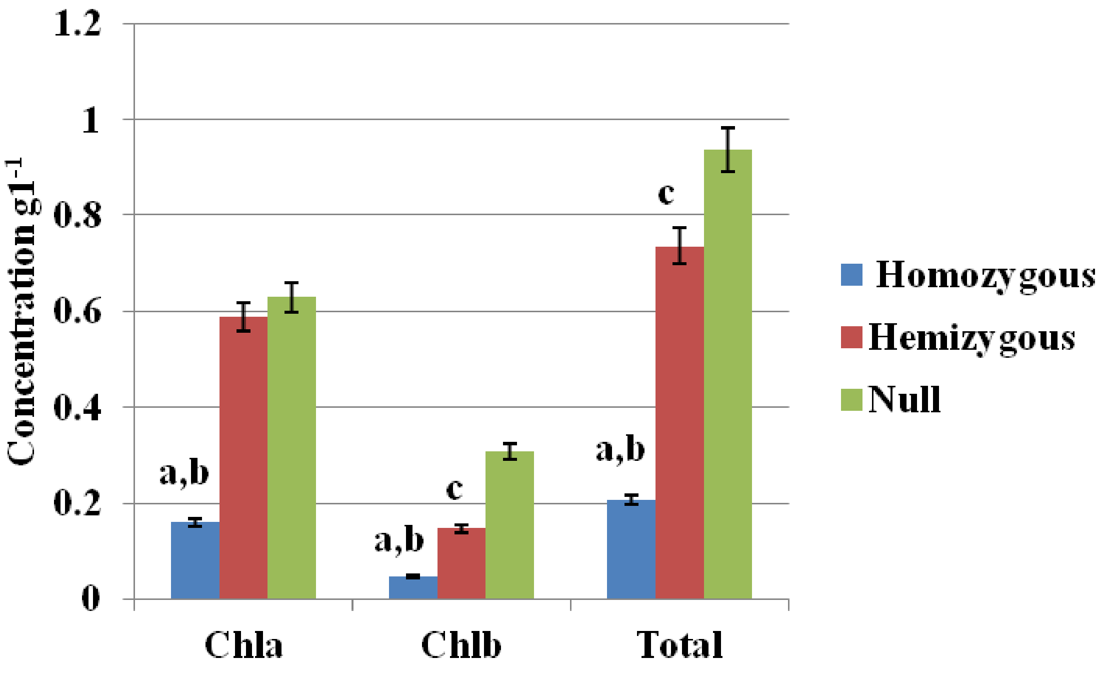

S3 Fig Relative concentration of chlorophyll pigments in the flag leaf tissue of the three groups of genotypes. The error bars represent SE, n=3. a refers to significant difference of homo from hemi, b significant difference of homo from null and c significant difference of hemi from null at P=0.05 significance level
