# Supplementary material for: Molecular and Functional Characterization of GR2-R1 Event Based Backcross Derived Lines of Golden Rice in the Genetic Background of a Mega Rice Variety Swarna
Source: PLoS One. 2017 Jan 9;12(1):e0169600. doi: 10.1371/journal.pone.0169600 (PMC5221763; doi:10.1371/journal.pone.0169600)
Supplement: S4 Fig — Primers were designed complementary to the ZmPsy sequence of the transgene. These primers when blasted to rice genome failed to show any hits and this indicate that the observed transcripts are not due to the amplification of endogenous rice Psy. The error bars represent SE, n = 3. (PDF) [file pone.0169600.s004.pdf]

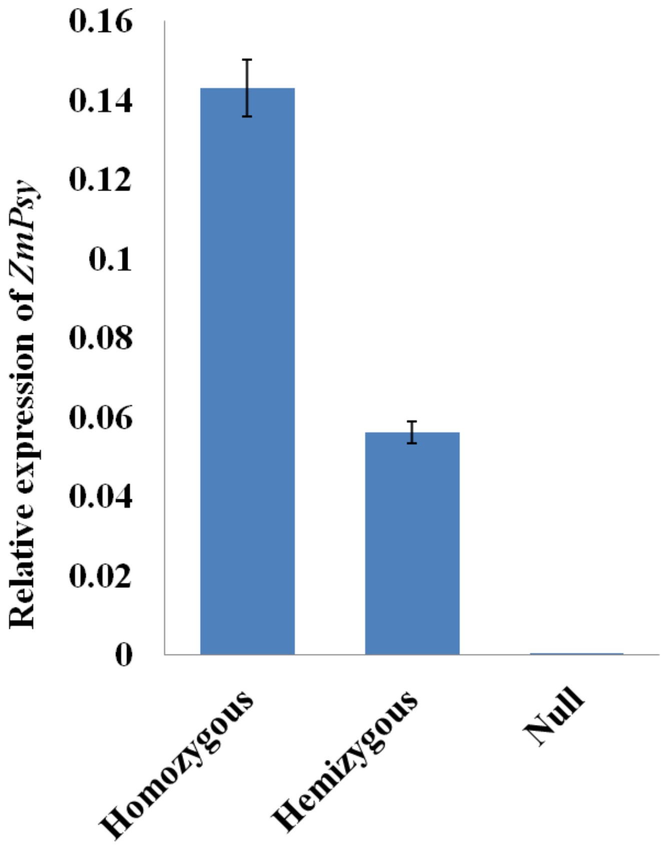

S4 Fig. Relative expression of *ZmPsy* in the leaf blades of homozygous, hemizygous and null lines. Primers were designed complementary to the *ZmPsy* sequence of the transgene. These primers when blasted to rice genome failed to show any hits and this indicate that the observed transcripts are not due to the amplification of endogenous rice *Psy*. The error bars represent SE, n=3
