# Supplementary material for: Molecular and Functional Characterization of GR2-R1 Event Based Backcross Derived Lines of Golden Rice in the Genetic Background of a Mega Rice Variety Swarna
Source: PLoS One. 2017 Jan 9;12(1):e0169600. doi: 10.1371/journal.pone.0169600 (PMC5221763; doi:10.1371/journal.pone.0169600)
Supplement: S5 Fig — The error bars represent SE, n = 3. (PDF) [file pone.0169600.s005.pdf]

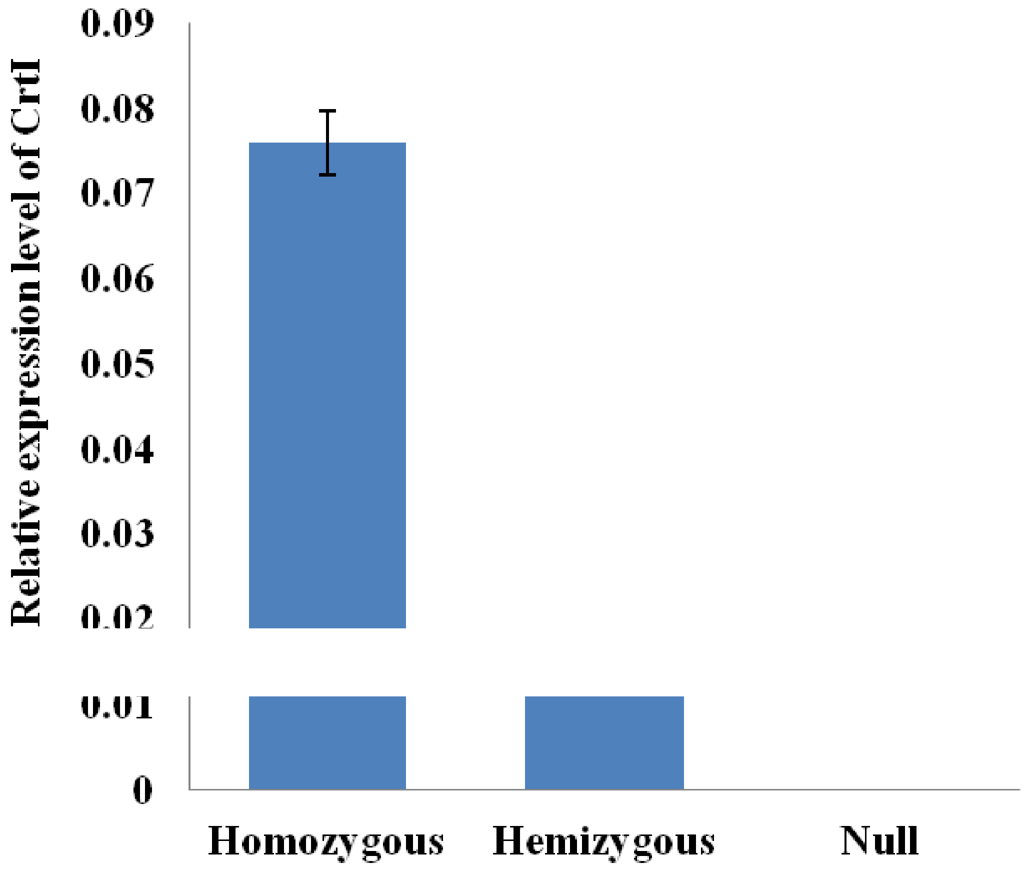

S5 Fig. Relative expression of *CrtI* in the leaf blades of homozygous, hemizygous and null lines. The error bars represent SE, n=3
