# Supplementary material for: Molecular and Functional Characterization of GR2-R1 Event Based Backcross Derived Lines of Golden Rice in the Genetic Background of a Mega Rice Variety Swarna
Source: PLoS One. 2017 Jan 9;12(1):e0169600. doi: 10.1371/journal.pone.0169600 (PMC5221763; doi:10.1371/journal.pone.0169600)
Supplement: S1 Table — (DOCX) [file pone.0169600.s006.docx]

S1 Table. Primer sequences used for expression analysis

| CrtI-Fp | CGCCAGGCGTTTTCTTTC |
| --- | --- |
| CrtI-Rp | AGGTGGCGAAGGGATTGC |
| ZmPSY1-Fp | AGATCTGTGAGGAGTATGCCAAGA |
| ZmPSY1-Rp | CGCCGCTCCTCTGTCATC |
| ZEP1-Fp | AACTTCCCTGTCCGTTTCCA |
| ZEP1-Rp | GGAACACGGCCTTTTTATCTGA |
| OsCPS-Fp | CGTACATCGACAGGATCATTAAGAA |
| OsCPS-Rp | AAAGATCGACCGGGTAAACGT |
| OsEUI-Fp | GGCTTGCTTTGGGAGTGATTAC |
| OsEUI-Rp | GCGAAGGGATGCTGAAGATG' |
| OsNCED-Fp | CGATTGTAGCTCAGATTGTTGTG |
| OsNCED-Rp | TGCCGCTGTATGTGAGCAT |
| OsGA20ox1-Fp | CCACTACAGGGCCGACATG |
| OsGA20ox1-Rp | GCAGGTGACGATGATGATTAAGC |
| Ubq-Fp | ACCACTTCGACCGCCACTACT |
| Ubq-Rp | ACGCCTAAGCCTGCTGGTT |
