# Supplementary material for: Molecular and Functional Characterization of GR2-R1 Event Based Backcross Derived Lines of Golden Rice in the Genetic Background of a Mega Rice Variety Swarna
Source: PLoS One. 2017 Jan 9;12(1):e0169600. doi: 10.1371/journal.pone.0169600 (PMC5221763; doi:10.1371/journal.pone.0169600)
Supplement: S2 Table — (DOCX) [file pone.0169600.s007.docx]

|  | **Homozygous** | **Hemizygous** | **Null** |
| --- | --- | --- | --- |
| Heading date | 104 days from  DOS | 95days from  DOS | 95days from  DOS |
| Plant Height (cm) | 47.2 ± 5.55 | 87.2 ± 10.6 | 90.9 ± 6.8 |
| Panicle length (cm) | 13.9 ± 3.2 | 26.2 ± 2.3 | 25.3 ± 2.7 |
| Per cent panicle unexerted | 46.2 ± 6.16 | 21.6 ± 4.98 | 26.4 ± 6.39 |
| Tiller Number | 18 ± 2.5 | 7.8 ± 1.8 | 9.8 ± 2.5 |
| No of effective tillers | 7 ± 2.5 | 7 ± 1.4 | 8 ± 1.9 |
| No. of Spikelets per panicle | 151.63 ± 18.8 | 245.6 ± 15.7 | 310.9±8.9 |
| Per cent spikelet fertility | 41.93 ± 15.4 | 90.79 ± 5.19 | 93.46 ±3.12 |
| 1000 seed weight (g) | 17.49 ± 0.13 | 16.65 ± 0.07 | 15.24 ± 0.17 |
| Yield per Plant (g) | 8.3±2.5 | 18.65±3.2 | 23.24±2.4 |

**S2 Table.** Agronomic performance of the backcross derived lines varying in their transgene zygosity in the background of mega rice variety Swarna
